# Characteristics and Treatment of Atrial Fibrillation with Respect to the Presence or Absence of Heart Failure. Insights from the Multicenter Polish Atrial Fibrillation (POL-AF) Registry

**DOI:** 10.3390/jcm10071341

**Published:** 2021-03-24

**Authors:** Monika Gawałko, Monika Budnik, Iwona Gorczyca, Olga Jelonek, Beata Uziębło-Życzkowska, Małgorzata Maciorowska, Maciej Wójcik, Robert Błaszczyk, Tomasz Tokarek, Renata Rajtar-Salwa, Jacek Bil, Michał Wojewódzki, Anna Szpotowicz, Małgorzata Krzciuk, Janusz Bednarski, Elwira Bakuła-Ostalska, Anna Tomaszuk-Kazberuk, Anna Szyszkowska, Marcin Wełnicki, Artur Mamcarz, Agnieszka Kapłon-Cieślicka

**Affiliations:** 1 1st Department of Cardiology, Medical University of Warsaw, 02-097 Warsaw, Poland; mgawalko@wum.edu.pl (M.G.); monibudnik@gmail.com (M.B.); 2 1st Clinic of Cardiology and Electrotherapy, Swietokrzyskie Cardiology Centre, 25-736 Kielce, Poland; iwona.goczyca@interia.pl (I.G.); olga_jelonek@wp.pl (O.J.); 3 Collegium Medicum, The Jan Kochanowski University, 25-369 Kielce, Poland; 4 Department of Cardiology and Internal Diseases, Military Institute of Medicine, 04-141 Warsaw, Poland; buzieblo-zyczkowska@wim.mil.pl (B.U.-Ż.); mmaciorowska@wim.mil.pl (M.M.); 5 Department of Cardiology, Medical University of Lublin, 20-059 Lublin, Poland; m.wojcik@umlub.pl (M.W.); robertblaszczyk1@wp.pl (R.B.); 6 Department of Cardiology and Cardiovascular Interventions, University Hospital, 30-688 Kraków, Poland; tomek.tokarek@gmail.com (T.T.); rajfura@op.pl (R.R.-S.); 7 Department of Invasive Cardiology, Centre of Postgraduate Medical Education, 02-507 Warsaw, Poland; biljacek@gmail.com (J.B.); michajerzywojewodzki@gmail.com (M.W.); 8 Department of Cardiology, Regional Hospital, 27-400 Ostrowiec Świętokrzyski, Poland; szpotowiczanna@wp.pl (A.S.); krzciukm@gazeta.pl (M.K.); 9 Department of Cardiology, St John Paul’s II Western Hospital, 05-825 Grodzisk Mazowiecki, Poland; medbed@wp.pl (J.B.); elwira.bakula@gmail.com (E.B.-O.); 10 Department of Cardiology, University Hospital of Białystok, 15-276 Białystok, Poland; a.tomaszuk@poczta.fm (A.T.-K.); annaszyszkowska92@gmail.com (A.S.); 11 3rd Department of Internal Diseases and Cardiology, Warsaw Medical University, 02-091 Warsaw, Poland; welnicki.marcin@gmail.com (M.W.); artur.mamcarz@wum.edu.pl (A.M.)

**Keywords:** atrial fibrillation, anticoagulation, heart failure

## Abstract

Background: We aimed to assess characteristics and treatment of AF patients with and without heart failure (HF). Methods: The prospective, observational Polish Atrial Fibrillation (POL-AF) Registry included consecutive patients with AF hospitalized in 10 Polish cardiology centers in 2019–2020. Results: Among 3999 AF patients, 2822 (71%) had HF (AF/HF group). Half of AF/HF patients had preserved ejection fraction (HFpEF). Compared to patients without HF (AF/non–HF), AF/HF patients were older, more often male, more often had permanent AF, and had more comorbidities. Of AF/HF patients, 98% had class I indications to oral anticoagulation (OAC). Still, 16% of patients were not treated with OAC at hospital admission, and 9%—at discharge (regardless of the presence of HF and its subtypes). Of patients not receiving OAC upon admission, 61% were prescribed OAC (most often apixaban) at discharge. AF/non–HF patients more often converted from AF at admission to sinus rhythm at discharge compared to AF/HF patients (55% vs. 30%), despite cardioversion performed as often in both groups. Class I antiarrhythmics were more often prescribed in AF/non–HF than in AF/HF group (13% vs. 8%), but still as many as 15% of HFpEF patients received them. Conclusions: Over 70% of hospitalized AF patients have coexisting HF. A significant number of AF patients does not receive the recommended OAC.

## 1. Introduction

Atrial fibrillation (AF) and heart failure (HF) are two colliding epidemics affecting approximately 1–2% of the world population [1], and resulting in significant morbidity and mortality [2,3]. HF affects overall more than 50% of patients with AF, whilst the prevalence of AF increases proportionally with the severity of the HF, reaching as much as over 50% of patients in New York Heart Association (NYHA) functional class IV [4]. HF and AF can cause and exacerbate each other through jointly shared risk factors, pathophysiology and mechanisms such as structural cardiac remodeling, activation of neurohormonal mechanisms, and rate-related impairment of left ventricular (LV) function [2].

The general approach to AF management does not differ between HF and other patients, with anticoagulation as the basis of treatment [2]. However, when it comes to maintenance of sinus rhythm and rate control, the matter becomes more complicated and the decision to adopt a treatment strategy depends on the patient’s age, HF etiology (tachycardia-related cardiomyopathy), AF duration and symptomatology, other coexisting cardiac and non-cardiac diseases and conditions, left atrial dimensions, anticipated adverse effects of antiarrhythmic drugs (AADs), and patient’s preferences [2].

There are significant differences in terms of pathophysiology, clinical features, and effectiveness of HF treatment depending on its phenotype i.e., HF with reduced ejection fraction (HFrEF), mid-range EF (HFmrEF), or preserved EF (HFpEF). In addition, diagnosis of HFpEF and HFmrEF in patients with AF is more challenging because elevation of natriuretic peptide levels and enlargement of the left atrium (which are diagnostic criteria for both HFmrEF and HFpEF) may be also associated with AF alone [2].

The aim of the study was to assess prevalence, clinical characteristics, and treatment of HF and its subtypes in hospitalized patients with AF.

## 2. Materials and Methods

### 2.1. Study Population

The POL-AF Registry (NCT04419012) was a prospective, observational study enrolling AF patients hospitalized in 10 cardiology departments (eight academic centers and two territorial centers) in Poland. Details on the study design and main results have been reported elsewhere [5,6]. Briefly, consecutive hospitalized patients in cardiology centers diagnosed with AF, except those admitted for AF ablation (in centers with electrotherapy labs), were included in the registry. Importantly, AF was not required to be the primary diagnosis and/or primary reason for index hospitalization, as the study included all hospitalized patients with AF diagnosis (except those admitted for AF ablation) to represent a broad spectrum of real-life AF patients. Patients with AF diagnosed upon hospital admission or during hospitalization were also included in the registry. Patients’ recruitment process started in January 2019 and lasted 12 months or longer, i.e., until the inclusion of 300 consecutive AF patients at each participating center (with the last patient enrolled in March 2020). Patients hospitalized several times during the study period were entered in the database under the same number. 

Diagnosis of AF and HF were made by attending physicians in accordance with the European Society of Cardiology (ESC) guidelines [7,8] In the current analysis, patients were categorized as having HF if they had a previous diagnosis of HF (classified as “previous HF diagnosis”) or were classified by the investigators as having HF with symptoms in NYHA class II, III, or IV during index hospitalization (classified as “HF de novo”). The methodology was similar to the one applied in previous studies [9,10,11,12,13,14,15,16]. Patients with HF and LV EF of <40%, 40–49%, and >50% were included in the HFrEF, HFmrEF, and HFpEF groups, respectively.

The study was approved by the Ethics Committee of the Swietokrzyska Medical Chamber in Kielce (104/2018). The Ethics Committee waived the requirement of obtaining informed consent from the patients. 

### 2.2. Data Collection

Data in the POL-AF Registry was gathered prospectively and included: demographics, medical history, electrocardiograms, results of laboratory tests (values on hospital admission), echocardiography, pharmacotherapy before hospital admission, and recommended at discharge.

### 2.3. Statistical Analysis

All continuous variables were tested for normality with the Kolmogorov–Smirnov test. Variables with normal distribution were expressed as mean ± standard deviation (SD). Nonparametric variables were expressed as median and interquartile range (IQR), and categorical variables as counts (*n*) with percentages (%). Fisher’s exact test (two group comparison) or chi-square test (three or more group comparison) were used to compare categorical variables. Differences in continuous parameters were compared using the Mann–Whitney U test (two group comparison) and the Kruskal–Wallis test (three groups comparison) in case of nonparametric variables and unpaired *t*-test (two group comparison) or ANOVA (three groups comparison) in case of parametric variables. To determine predictors of non-prescription of oral anticoagulation (OAC) in AF/non–HF and AF/HF groups, multiple logistic regression analysis, using the stepwise forward procedure, was performed, including following variables: age ≥ 75 years, female sex (vs. male), LV EF < 50% (for the AF/HF group), hypertension, vascular disease (including those hospitalized for acute coronary syndrome for the analysis at discharge), diabetes, previous stroke, previous hemorrhagic events, renal dysfunction (chronic kidney dysfunction for the analysis at hospital admission, and glomerular filtration rate (GFR) < 60 mL/min/1.73m^2^ for the analysis at discharge), liver disease, anemia (hemoglobin < 12 g/dL for women and <13 g/dL for men), antiplatelet therapy (at hospital admission and at hospital discharge for admission and discharge analyses, respectively), alcohol overconsumption, and chronic treatment with nonsteroidal anti-inflammatory drugs (NSAIDs). A two-sided *p* value of 0.05 was considered statistically significant. For database management and statistical analysis, we used SAS Institute Inc. 2015. SAS/IML^®^ 14.1 User’s Guide (SAS Institute Inc. Cary, NC, USA).

## 3. Results

### 3.1. Study Population

**Figure 1:**
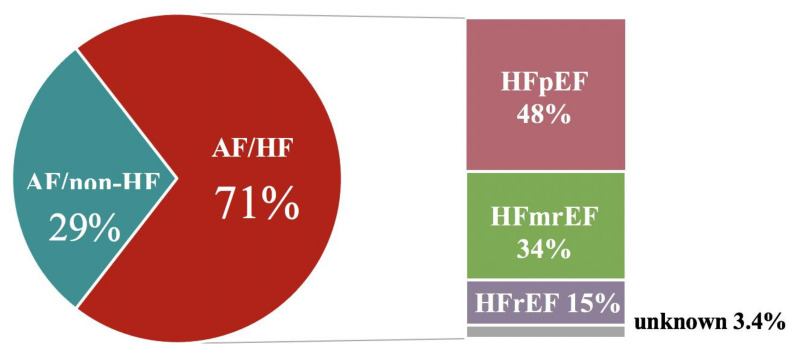
Frequency of heart failure and its subtypes in hospitalized atrial fibrillation patients. Abbreviations: AF, atrial fibrillation; HF, heart failure; HFmEF, heart failure with mild-range ejection fraction; HFpEF, heart failure with preserved ejection fraction; HFrEF, heart failure with reduced ejection fraction.

Overall, 3999 patients were enrolled in the POL-AF Registry. A total of 3396 patients (85%) were enrolled in academic centers and 603 patients (15%)—in territorial centers. Among them, 2822 (71%) had a diagnosis of HF, that was confirmed by previous documentation in 2621 (93%) and was first made during index hospitalization in 201 (7.1%) patients (Table 1). Of those, 950 (34%) had HFrEF, 417 (15%)—HFmrEF, 1359 (48%)—HFpEF and for 96 (3.4%) there were no information on LV EF and/or HF subtype in the registry database (Figure 1).

### 3.2. Atrial Fibrillation Patients with and without Heart Failure

Clinical and laboratory characteristics of the study groups are presented in Table 1 and Table 2 and Table S1 (Supplementary Materials online), respectively.

In patients with AF and no HF (AF/non–HF), median age was 70 years, half were female, 56% had paroxysmal AF, 80% had hypertension, 37% had vascular disease, 13% had previous thromboembolic events, 5% had previous hemorrhagic events, and the median CHA_2_DS_2_-VASc score was 3, with 78% of patients with class I indications to OAC. Compared to AF/non–HF patients, those with both AF and HF diagnosis (AF/HF) were older (median age 74 years), more often male, more often had permanent AF (34% vs. 15%), and had an even higher prevalence of comorbidities, including hypertension, vascular disease, diabetes, and previous thromboembolic (18%) and hemorrhagic events (7%), hence were at higher thromboembolic risk based on the CHA_2_DS_2_-VASc score (5 points), with 98% of patients with class I indications to OAC. 

The most common primary reason for index hospitalization in AF/non–HF patients was direct current cardioversion (23%). The most common primary reason for index hospitalization in the AF/HF group was HF decompensation (29%). Direct current cardioversion in AF/HF was as commonly performed as in AF/non–HF (22% vs. 23%, *p* = 0.74) (Table 1). 

At hospital admission, 66% of AF/non–HF and 76% of AF/HF patients were in AF. If in AF at hospital admission, AF/non–HF patients more often converted to sinus rhythm at discharge as compared to AF/HF patients (55% vs. 30%, *p* < 0.05), given higher prevalence of permanent AF in AF/HF group. Irrespective of HF, the majority of patients with sinus rhythm on an electrocardiogram at hospital admission remained in sinus rhythm at discharge (99% of AF/non–HF and 97% of AF/HF patients, *p* > 0.05) (Figure S1; Supplementary Materials online).

**Figure 2:**
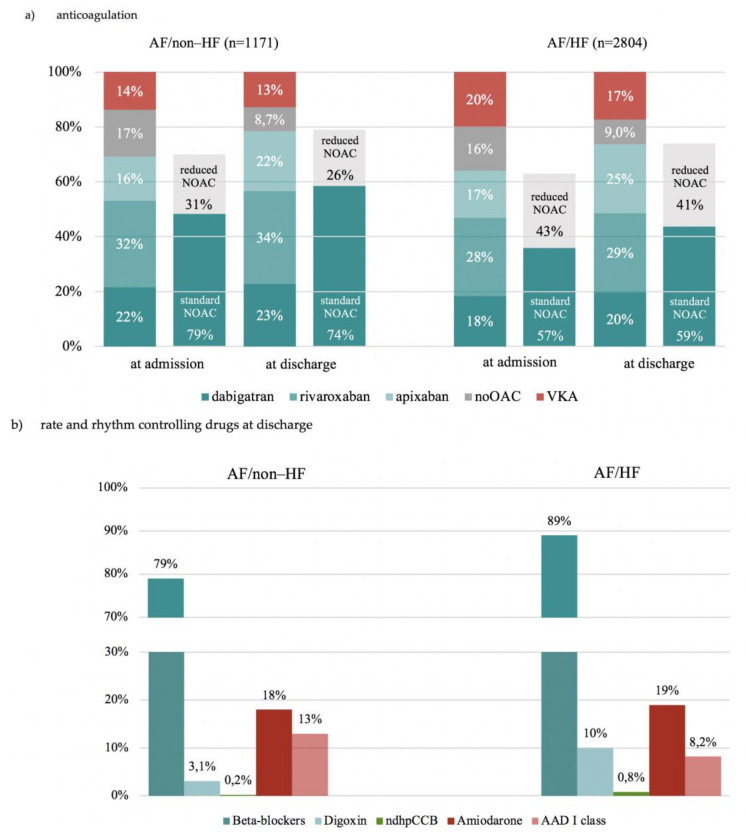
Pharmacotherapy of hospitalized atrial fibrillation patients depending on presence or absence of heart failure and its subtypes. (**a**) Differences between AF/non–HF vs. AF/HF group were statistically significant for all treatment subgroups (*p* < 0.05), except no OAC at baseline (*p* = 0.64), apixaban treatment at baseline (*p* = 0.29), and no OAC (*p* = 1.00) at discharge. Differences between AF/non–HF vs. AF/HF group regarding reduced and standard NOAC doses were statistically significant (*p* < 0.05). (**b**) Dronedarone was not prescribed in any of the groups. Differences in pharmacotherapy between AF/nonHF vs. AF/HF group were statistically significant (*p* < 0.05), except amiodarone treatment (*p* = 0.53). Abbreviations: See Table 1; AAD, antiarrhythmic drug; ndhpCCB, non-dihydropyridine calcium channel blockers, NOAC, non-vitamin K antagonist oral anticoagulant.

At hospital admission, 17% of AF/non–HF and 16% of AF/HF patients, did not receive any anticoagulation (Figure 2). 

**Figure 3:**
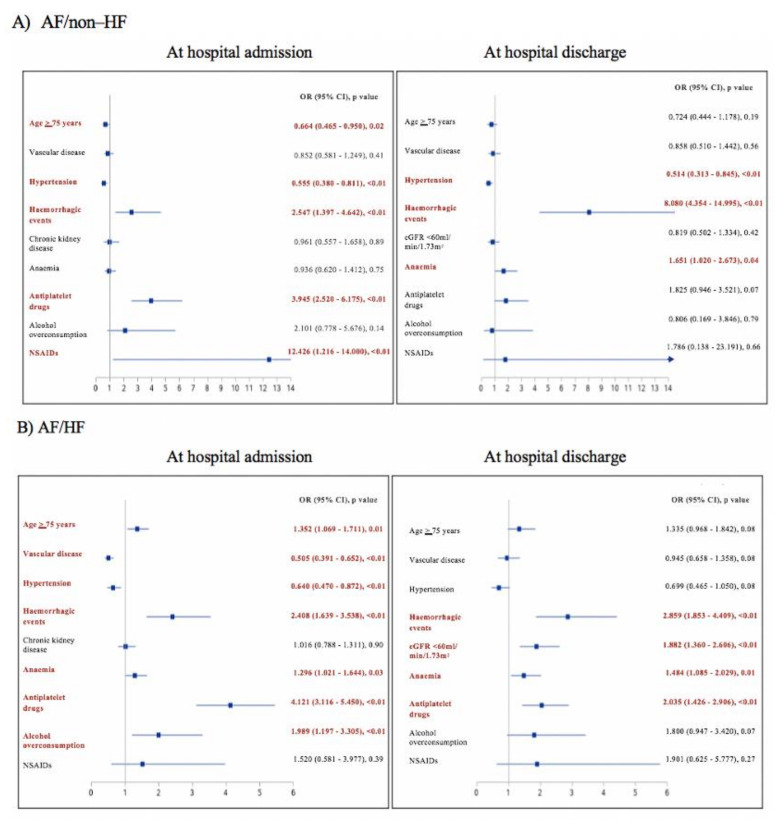
Predictors of non-prescription of oral anticoagulation in atrial fibrillation patients without heart failure (**A**) and with heart failure (**B**). Following variables were included in analysis: age ≥ 75 years, female sex (vs male), LV EF < 50% (for the AF/HF group), hypertension, vascular disease (including those hospitalized for acute coronary syndrome for the analysis at discharge), diabetes, previous stroke, previous hemorrhagic events, renal dysfunction (chronic kidney dysfunction for the analysis at hospital admission, and GFR < 60 mL/min/1.73 m^2^ for the analysis at discharge), liver disease, anemia (hemoglobin < 12 g/dL for women and <13 g/dL for men), antiplatelet therapy (at hospital admission and at hospital discharge for admission and discharge analyses, respectively), alcohol overconsumption and chronic treatment with NSAIDs. Abbreviations: See Table 1; CI, coincidence interval; NSAIDs, non-steroidal anti-inflammatory drugs; OR, odds ratio.

A total of 69% of such patients in the AF/non–HF group and 96% of such patients in the AF/HF group had class I indications to OAC (Figure S2, Supplementary Materials online). Of patients not receiving OAC upon hospital admission, 58% in the AF/non–HF group and 63% in the AF/HF group were prescribed OAC (most often apixaban) at hospital discharge (Table S2, Supplementary Materials online). Conversely, of AF/non–HF patients with no indications to OAC, almost three quarters received OAC at hospital admission (Table 3). Predictors of non-prescription of OAC in both groups are shown in Figure 3.

Irrespective of the presence of HF, majority of patients were prescribed non-vitamin K antagonist OAC (NOAC) with a predominance of rivaroxaban (Figure 2a). However, apixaban was the type of OAC most frequently initiated during hospitalization in both AF/HF and AF/non–HF group. (Figure 2a; Table S2; Supplementary Materials online). Reduced NOAC doses were more often prescribed in AF/HF group both at baseline (43% vs. 31%) and at discharge (41% vs. 26%) as compared to AF/non–HF group (Figure 2a). 

At hospital discharge, beta-blockers were the most commonly prescribed medications for rhythm/rate control in both groups (79% in AF/non–HF, and 89% in AF/HF group). Digoxin was more often prescribed in the AF/HF group (10% vs. 3.1%). Non-dihydropyridine calcium channel blockers (CCB) were rarely prescribed in either group. Amiodarone was as frequently prescribed in both groups (18% and 19%). Class I antiarrhythmic drugs (AADs) were more often prescribed in AF/non–HF than in AF/HF group (13% vs. 8%) (Figure 2b). None of the patients received dronedarone.

Diuretics (73% vs. 45%), renin-angiotensin system (RAS) inhibitors (81% vs. 71%), and mineralocorticoid receptor antagonists (50% vs. 17%) were more often prescribed in AF/HF patients as compared to AF/non–HF patients (Figure S3; Supplementary Materials online).

### 3.3. Atrial Fibrillation Patients with Heart Failure Depending on Ejection Fraction

The comparison of clinical and laboratory characteristics of AF patients with HFrEF, HFmrEF and HFpEF are shown in Table 1 and Table 2 and Table S1, respectively. Pharmacotherapy of those patients is presented in Figures S3 and S4 (Supplementary Materials online). All HF subgroups were most often prescribed rivaroxaban, with the exception of HFrEF patients at discharge who were more often prescribed apixaban. The frequency of apixaban prescription increased, and that of rivaroxaban decreased with decreasing LVEF. There were no other differences in terms of OAC treatment between HF subtypes (Figure S4a). Reduced doses of NOACs were more often prescribed in HFrEF at baseline (Figure S4b). Beta-blockers were the most commonly prescribed medications for rhythm/rate control in all groups, with no differences between HF subtypes. Digoxin and amiodarone were more often prescribed in the HFrEF group. Noteworthy, 15% of HFpEF were prescribed AADs class I (Figure S4c). No statistically significant difference was observed in prescription of RAS inhibitors between HF subgroups. Patients with HFrEF more often received diuretics and mineralocorticoid receptor antagonists, whereas patients with HFpEF were more often treated with CCBs as compared to other subgroups (Figure S3).

## 4. Discussion

The main advantage of registries is their observational character, which allows one to study real-world, unselected groups of patients encountered in everyday clinical practice. The POL-AF registry included AF patients hospitalized in cardiology centers and, thus, it does not reflect the characteristics of the general AF population. Still, given the large number of consecutive patients enrolled in the registry, irrespective of the reason for index hospitalization or the presence of AF at hospital admission, the POL-AF registry provides a reliable description of this specific AF subpopulation.

The most important findings of our study are as follows: (1) Over 70% of AF patients hospitalized in cardiology centers had coexisting HF, mostly HFpEF (2); due to advanced age and high comorbidity burden AF/HF patients had a high CHA_2_DS_2_-VASc score (median: 5 points); with 98% of patients with class I indications to OAC (3); however, at hospital admission, 16% of AF/HF patients did not receive any OAC (4); predictors of OAC non-prescription in patients with AF and HF included age ≥ 75 years, previous hemorrhagic events, renal dysfunction, anemia, antiplatelet therapy and alcohol overconsumption; and (5) 15% of AF patients with HFpEF were treated with class I AADs, despite a diagnosis of structural heart disease.

The prevalence of HF in the POL-AF population was higher than reported in previous studies [9,10,11,12,13,14,15,16]. This may be explained by the fact that the POL-AF registry included AF patients hospitalized in cardiology centers, as well as by the fact that previous studies reported mostly HF with moderately or severely reduced LV EF [15,16], while in POL-AF, HFpEF constituted half of all HF cases. This reflects the close relationship between HFpEF and AF, resulting not only from increased left atrial pressures in the course of HF, but also from shared risk factors of these two clinical entities. Consequently, the prevalence of AF in HFpEF is even higher than in HFrEF [17,18]. In the ESC-HF Long-Term registry, the prevalence of HFpEF in patients hospitalized for HF was 29%, while in our study, in AF/HF patients, it was much higher (48%), which further proves the strong association of AF with HFpEF [19,20]. The diagnosis of HFpEF in patients with AF may be problematic because of the difficulty in separating symptoms that are due to HF from those due to AF [21]. Natriuretic peptides are elevated, and left atrial dilatation is common in AF regardless of concomitant HF [22,23]. This issue has been addressed in the recent consensus recommendation from the Heart Failure Association of the ESC, with higher cut-offs for HFpEF diagnosis for both left atrial volume index and natriuretic peptides in AF patients in the HFA-PEFF score [22]. On the other hand, AF is highly prevalent in HFpEF, even more prevalent than in HFrEF [17,18], and presence of AF was actually proven to predict HFpEF [24]. In the H2FPEF score, a modern score to predict HFpEF, derived from a population with HFpEF confirmed with a gold standard, i.e., invasive hemodynamic exercise testing, presence of AF is the strongest predictive factor for HFpEF [24]. High prevalence of AF in HFpEF patients results not only from a HF-related elevation in left atrial pressure, but also from a common pathophysiological background of both AF and HFpEF, which share the same risk factors, including older age, hypertension, obesity, metabolic syndrome and other cardiac and extra-cardiac comorbidities. Thus, high prevalence of HFpEF in the AF population in our study is not surprising, even if the finding is, itself, novel.

Our study performed a thorough analysis of patients with AF and HFmrEF. The ESC guidelines do not give specific recommendations for management of HFmrEF, but they suggest that, since patients with HFmrEF have mostly been included in trials of HFpEF, rather than HFrEF, they should be treated with the same management principle as patients with the former, until new evidence is available [7]. In current clinical practice, compared with HFrEF patients, fewer patients with HFpEF and HFmrEF appear to receive diuretics, beta-blockers, mineralocorticoid receptor antagonists, and RAS inhibitors [7,25]. However, in our study there was no difference in the number prescribed the aforementioned drugs between subgroups of HF except MRAs and diuretics. Further randomized clinical trials with long-term follow-up of this group are required before particular treatment strategies in AF patients with HFmrEF can be recommended.

Indeed, in the 7.1% of patients with “de novo” HF diagnosis, an unequivocal distinction between AF-related dyspnea and AF associated with HF may not be possible, especially in patients with HFpEF. However, the resolution of symptoms after conversion to sinus rhythm suggests AF-related dyspnea, while their persistence despite conversion to sinus rhythm (in patients fulfilling other HF diagnostic criteria) confirms correct HF diagnosis. As presented in Figure S1b, 55% of patients with AF at hospital admission converted to sinus rhythm during hospitalization, which might have helped their attending physicians in securing a correct HF diagnosis. Furthermore, 29% of HF patients were in sinus rhythm (and not AF) on hospital admission (Figure S1a), meaning that their symptoms on admission were attributable to HF, and not AF. 

The background etiology and epidemiology differ between the particular types of HF and our results reflect previous observations [26,27,28]. Age and comorbidity burden were high even in the AF/non–HF group. AF/HF patients, as expected, had even more comorbidities. Median CHA_2_DS_2_-VASc scores were 3 and 5 in AF/non–HF and AF/HF groups, respectively. Despite the majority of patients with previous diagnosis of AF and class I indications to OAC, a significant proportion of patients in both groups did not receive OAC upon hospital admission. This is somewhat similar to the results of our previous study of AF patients admitted for AF direct current cardioversion or AF ablation in years 2012–2016, where also 17% of patients were not treated with any OAC, although it must be noted that these two populations were very different [29]. Low prescription of recommended OAC is complex and may compounded by many factors. In our study, predictors of OAC non-prescription in both AF/non–HF and AF/HF groups included age >75 years, previous hemorrhagic events, hypertension and antiplatelet therapy at hospital admission, and hemorrhagic events and anemia at hospital discharge. This variety of factors associated with OAC non-prescription is line with previous studies [30]. Future efforts to characterize reasons for non-prescription and determine whether educational or quality improvement interventions will increase OAC utilization in AF patients are warranted.

More than half of patients (61%) in the current study were ultimately discharged on OAC, mainly on apixaban. This could be explained by recent data implying superiority of apixaban over other NOACs. Compared to VKA, all NOACs are associated with fewer cardiovascular events, including myocardial infraction and stroke in patients with both AF and HF based on the recent study by Amin et al. [31]. The study reported that AF/HF patients prescribed NOAC had 36% lower odds of stroke/systemic embolism, 34% lower odds of major bleeding and 27% lower odds of major adverse cardiovascular events compared to VKA. Moreover, when apixaban users were compared to patients taking rivaroxaban and dabigatran, apixaban showed better results. Those patients had a 45% lower risk of bleeding and a 14% lower risk of major adverse cardiovascular events versus rivaroxaban, and corresponding risk reductions compared to dabigatran were 29% and 20% [31]. However, recent studies have shown inconclusive results regarding the superiority of one NOAC over others in subgroup populations including elderly patients (≥85-year-old) [32] or those with high prevalence of prescribed drugs interacting with NOAC pharmacokinetics [33].

In our study, RAS inhibitors were frequently used, irrespective of HF presence, which is not surprising given the high prevalence of coexisting hypertension, coronary artery disease, diabetes, and renal dysfunction in both AF/HF and AF/non–HF groups. This is in line with the most recent ESC AF guidelines [34], recommending comprehensive AF treatment consisting of three main pillars, anticoagulation (A), better symptom control (B), and comorbidities and risk factors control (C). High frequency of treatment with RAS inhibitors (concordant with the “C” element) suggests that this time the guidelines followed clinical practice, as our registry was conducted before the introduction of the 2020 ESC AF guidelines [34].

In the AF Follow-up Investigation of Rhythm Management (AFFIRM) trial, it was demonstrated that absence of HF favored the rate control strategy, but no differences were seen in patients with HF [35]. Further, Atrial Fibrillation and Congestive Heart Failure (AF-CHF) investigators indicated no differences between the rate and rhythm control strategy in AF patients with HF with regard to all-cause death, stroke and worsening HF, however AF hospitalization risk in the rhythm control group was higher than that in the rate control group [36]. On the other hand, catheter ablation was proved to improve quality of life, symptoms, and LV function [37] and reduce all-cause mortality and hospitalization [38] in other randomized control trials. A recent substudy of a meta-analysis comparing catheter ablation and rate control strategy, reported no differences in the composite of all-cause mortality and HF readmission between the two groups. However, when compared with rate control, catheter ablation was associated with improvement in LV function and health-related quality of life [39]. Still, superiority of rhythm over rate control still needs to be confirmed in large randomized controlled trials. In our study, beta-blockers were the most common rhythm/rate control drugs in both AF/non–HF and AF/HF patients. Beta-blockers are known to prolong life in HFrEF patients who are in sinus rhythm [40], however, their use has been questioned to improve prognosis in HFrEF and AF [41]. Still, in our study, most patients with AF and HFrEF were prescribed beta-blockers. Digoxin was rarely used, especially in AF/non–HF patients, even though many of them were elderly. This could be explained by heterogenous data regarding treatment with digoxin. Observational studies have associated digoxin use with excess mortality in AF patients [42,43,44]. However, recent metanalysis reported neutral effect on mortality and a lower rate of hospital admissions on digoxin treatment compared to placebo and emphasized that all reported adverse outcomes associated with digoxin were more likely due to selection and prescription biases rather than harm caused by digoxin [45]. Recent results from the Rate Control Therapy Evaluation in Permanent Atrial Fibrillation (RATE-AF) trial, confirmed safety of digoxin in AF/HF patients, where it was safer and more effective than beta-blockers [46]. Although amiodarone is associated with serious long-term side-effects [47,48], and is thus considered a second-line antiarrhythmic in AF patients without HF, almost one-fifth of AF/non–HF patients received amiodarone, which was as often as in the AF/HF group. In AF/non–HF patients, amiodarone was prescribed more frequently than AADs class I. This may be to some extent explained by a high prevalence of coronary artery disease in those AF/non–HF patients (62% of patients prescribed amiodarone had coronary artery disease). In the AF/HF group, amiodarone might have also been prescribed for indications other than AF (such as ventricular arrhythmias). Finally, it is surprising that AADs class I were used in 8% of HF patients, including 15% of patients diagnosed with HFpEF, despite AADs class I being contraindicated in patients with known structural heart disease such as heart failure, hypertrophic cardiomyopathy and valvular heart disease [2,49]. The proportion of patients with HFpEF receiving contraindicated AADs class I was even higher in patients hospitalized in academic centers (16% vs. 5.3% in territorial centers, *p* < 0.01) However, our data must be interpreted in relation to the studied population, in which 85% were patients hospitalized in academic centers. This means that the percentage of patients not receiving OAC despite indications or receiving antiarrhythmics class I despite contraindications may be even higher in territorial hospitals, given the differences in characteristics and treatment between patients hospitalized in academic vs. territorial hospitals in the POL-AF registry (Table S3; Supplementary Materials online).

### Limitations

The limitations of our study arise largely from the type of data analyzed (i.e., registry-derived). First, there was a certain proportion of data missing for some of the patients. Thus, we showed the number of patients for whom data were available in each table and figure. Second, only data predefined by the coordinators of the POL-AF registry were gathered in the database. Those did not include concentrations of natriuretic peptides or echocardiographic indices of LV diastolic function as well as HF etiology. Therefore, definitive verification of the pertinence of HFpEF diagnosis was not possible as well as definitively determining whether the patient had HF or AF first. However, the registry was conducted in academic and territorial centers with experience in managing multicenter registries and clinical trials, and investigators were requested to verify both AF and HF diagnosis in each patient according to the current guidelines [2,7]. Third, 85% of patients were enrolled in academic centers, which is important for data interpretation. Last, patients referred for catheter ablation for AF (pulmonary vein isolation) were excluded from the registry. Exclusion of patients referred for ablation was done in order to avoid selection bias, given that many academic cardiology centers perform catheter ablations, and AF patients admitted for ablation are mostly a specific group of young patients with no or few comorbidities. Given a high number of academic centers with an electrophysiology lab in the POL-AF registry, the number of young patients admitted for ablation would be high, and inclusion of such patients would artificially lower the age of the studied population and decrease the number of comorbidities as well as both thromboembolic and bleeding risks. This would then not properly reflect the characteristics of hospitalized AF patients who are mostly elderly with many comorbidities. Furthermore, patients referred for ablation are usually referred to an academic hospital from all over the region, while patients admitted for other elective procedures (such as cardioversion) or for acute reasons are mostly local residents of the area in which a given hospital (academic or territorial) is situated. On the other hand, it needs to be emphasized that the population of the POL-AF registry represents hospitalized patients with AF and not a general population of AF patients. 

## 5. Conclusions

Herein, we performed a thorough analysis of patients with AF and HF subtypes including HFpEF, HFmrEF, and HFrEF. Almost all of the AF/HF patients had class I indications to OAC. Still, one in six AF patients did not receive OAC at hospital admission, irrespective of the presence of HF. Similarly, one in six HFpEF patients with AF was treated with class I AADs, despite a diagnosis of structural heart disease. Our study provides clinical characteristics and description of real-life treatment of AF/HF patients, showing some discrepancy between current guidelines and real-life practice.

## Figures and Tables

**Table 1:** Baseline characteristics of hospitalized atrial fibrillation patients depending on presence or absence of heart failure and its subtypes.

Variable	AF/non–HF (*n* = 1177)	AF/HF (*n* = 2822)	*p* ^1^	AF/HF with Known EF (*n* = 2726)			*p* ^2^
				HFrEF (*n* = 950)	HFmrEF (*n* = 417)	HFpEF (*n* = 1359)	
Demographics							
Age (years)	70.0 (64.0–78.0)	74.0 (66.0–82.0)	<0.01	71 (63–80)	76 (61–83)	75 (67–82)	<0.01
Females (%)	540 (50%)	1164 (41%)	<0.01	269 (29%)	163 (40%)	629 (50%)	<0.01
BMI (kg/m^2^)	28.6 (26.0–31.3) *n* = 677	28.4 (25.6–32.4) *n* = 2065	0.54	28.1 (25.2–32.2) *n* = 633	29.0 (25.8–32.8) *n* = 308	28.6 (25.7–32.4) *n* = 1087	0.40
Primary reason of index hospital admission							
AF without any procedures	159 (14%)	93 (3.3%)	<0.01	19 (2.0%)	13 (3.1%)	57 (4.2%)	0.01
DC cardioversion for AF	267 (23%)	626 (22%)	0.74	105 (11%)	78 (19%)	438 (32%)	<0.01
HF decompensation	NA	806 (29%)	NA	380 (40%)	118 (28%)	296 (22%)	<0.01
Elective CIED * implantation/replacement	130 (11%)	230 (8.2%)	<0.01	92 (9.7%)	36 (8.6%)	94 (6.9%)	0.053
ACS	45 (3.8%)	202 (7.2%)	<0.01	80 (8.4%)	45 (11%)	73 (5.4%)	<0.01
Elective PCI	91 (7.7%)	292 (10%)	0.01	101 (11%)	50 (12%)	122 (9.0%)	0.15
Non-AF-ablation	78 (6.6%)	132 (4.7%)	0.02	34 (3.6%)	18 (4.3%)	76 (5.6%)	0.07
Other	388 (33%)	441 (16%)	<0.01	139 (15%)	59 (14%)	203 (15%)	0.92
AF type							
AF paroxysmal	664 (56%)	1259 (45%)	<0.01	352 (37%)	149 (36%)	719 (53%)	<0.01
AF persistent	337 (29%)	596 (21%)	<0.01	204 (22%)	103 (25%)	277 (20%)	0.17
AF permanent	176 (15%)	967 (34%)	<0.01	394 (42%)	165 (40%)	363 (26%)	<0.01
AF history							
Prior AF history	1043 (89%)	2654 (94%)	<0.01	893 (94%)	379 (91%)	1287 (95%)	0.02
Prior DC cardioversion for AF	211 (18%)	709 (25%)	<0.01	146 (15%)	93 (22%)	460 (34%)	<0.01
Prior AF-ablation	104 (8.8%)	160 (5.7%)	<0.01	44 (4.6%)	23 (5.5%)	90 (6.6%)	0.13
EHRA I	288 (38%) *n* = 753	1067 (53%) *n* = 2027	<0.01	292 (45%) *n* = 652	139 (48%) *n* = 291	602 (59%) *n* = 1023	<0.01
EHRA II	353 (47%) *n* = 753	614 (30%) *n* = 2027	<0.01	228 (35%) *n* = 652	96 (33%) *n* = 291	265 (26%) *n* = 1023	<0.01
-EHRA IIa	148 (20%) *n* = 753	246 (12%) *n* = 2025	<0.01	84 (13%) *n* = 652	44 (15%) *n* = 290	108 (11%) *n* = 1022	0.07
-EHRA IIb	113 (15%) *n* = 753	223 (11%) *n* = 2025	<0.01	70 (11%) *n* = 652	37 (13%) *n* = 290	114 (11%) *n* = 1022	0.66
EHRA III	96 (12%) *n* = 753	281 (14%) *n* = 2027	0.50	101 (15%) *n* = 652	46 (16%) *n* = 291	133 (13%) *n* = 1023	0.26
EHRA IV	16 (2.1%) *n* = 753	65 (3.2%) *n* = 2027	0.16	31 (4.8%) *n* = 652	10 (3.4%) *n* = 291	23 (2.3%) *n* = 1023	0.02
HF							
Previous HF diagnosis	NA	2621 (93%)	NA	936 (99%)	394 (94%)	1214 (89%)	<0.01
HF de novo	NA	201 (7.1%)	NA	14 (1.5%)	23 (5.5%)	145 (11%)	<0.01
NYHA I/II at admission	NA	1473 (55%) *n* = 2665	NA	327 (37%) *n* = 889	207 (53%) *n* = 392	886 (68%) *n* = 1301	<0.01
NYHA III at admission	NA	859 (32%) *n* = 2665	NA	398 (45%) *n* = 889	138 (35%) *n* = 392	304 (23%) *n* = 1301	<0.01
NYHA IV at admission	NA	190 (7.1%) *n* = 2665	NA	115 (13%) *n* = 889	25 (6.4%) *n* = 392	45 (3.5%) *n* = 1301	<0.01
Comorbidities							
Hypertension	937 (80%)	2407 (85%)	<0.01	761 (80%)	349 (84%)	1216 (90%)	<0.01
Vascular disease	434 (37%)	1811 (64%)	<0.01	660 (69%)	291(70%)	798 (59%)	<0.01
Previous stroke	120 (10%)	380 (13%)	<0.01	133 (14%)	58 (14%)	171 (13%)	0.60
Thromboembolic events	151 (13%)	508 (18%)	<0.01	167 (18%)	66 (16%)	254 (19%)	0.39
Hemorrhagic events	58 (4.9%)	193 (6.8%)	0.02	68 (7.2%)	30 (7.2%)	85 (6.3%)	0.63
Diabetes mellitus	319 (27%)	1047 (37%)	<0.01	397 (42%)	158 (38%)	450 (33%)	<0.01
Chronic kidney disease	138 (12%)	891 (32%)	<0.01	346 (36%)	126 (30%)	384 (29%)	<0.01
Smoking (current/former)	256 (23%) *n* = 1098	795 (30%) *n* = 2677	<0.01	332 (37%) *n* = 904	106 (27%) *n* = 391	322 (25%) *n* = 1296	<0.01
Alcohol overconsumption (≥8 drinks/week)	21 (1.9%) *n* = 1107	129 (4.8%) *n* = 2701	<0.01	68 (7.5%) *n* = 906	13 (3.3%) *n* = 395	44 (3.4%) *n* = 1312	<0.01
Liver disease	46 (3.9%)	215 (7.6%)	<0.01	103 (11%)	31 (7.4%)	74 (5.5%)	<0.01
Thyroid disease	205 (17%)	522 (19%)	0.44	159 (17%)	91 (22%)	257 (19%)	0.08
COPD/asthma	67 (5.7%)	381 (14%)	<0.01	136 (14%)	49 (12%)	182 (13%)	0.44
CIED therapy *	162 (14%)	717 (25%)	<0.01	343 (36%)	95 (23%)	255 (19%)	<0.01

^1^*p* value for difference between patients with and without heart failure. ^2^ *p* value for difference between heart failure patients with reduced, mid-range, and preserved ejection fraction. * defined as use of pacemaker, implantable cardioverter-defibrillator and/or cardiac resynchronization therapy. Abbreviations: AF, atrial fibrillation; BMI, body mass index; CAD, coronary artery disease; CIED; cardiac implantable electronic device; COPD, chronic obstructive pulmonary disease; DC, direct current; EHRA, European Heart Rhythm Association; HF, heart failure; HFmEF, heart failure with mild-range ejection fraction; HFpEF, heart failure with preserved ejection fraction; HFrEF, heart failure with reduced ejection fraction; NA, non-applicable; NYHA, New York Heart Association; PAD, peripheral artery disease.

**Table 2:** Thromboembolic and bleeding risk of hospitalized atrial fibrillation patients depending on presence or absence of heart failure and its subtypes.

Variable	AF/non–HF (*n* = 1177)	AF/HF (*n* = 2822)	*p* ^1^	AF/HF with Known EF (*n* = 2726)			*p* ^2^
				HFrEF (*n* = 950)	HFmrEF (*n* = 417)	HFpEF (*n* = 1359)	
CHA2DS2-VASc score	3 (2–4) 3.2 ± 1.7	5 (4–6) 4.9 ± 1.6	<0.01	5 (4–6) 4.7 ± 1.7	5 (4–6) 5.0 ± 1.6	5 (4–6) 5.0 ± 1.5	<0.01
No indications to OAC ^3^	89 (7.6%)	0 (0%)	<0.01	0 (0%)	0 (0%)	0 (0%)	1.00
Class IIa indications to OAC ^4^	174 (15%)	61 (2.2%)	<0.01	36 (3.8%)	5 (1.2%)	18 (1.3%)	<0.01
Class I indications to OAC ^5^	914 (78%)	2761 (98%)	<0.01	914 (96%)	412 (99%)	1341 (99%)	<0.01
HAS-BLED score	2 (1–2) 1.9 ± 0.9	2 (2–3) 2.2 ± 0.9	<0.01	2 (2–3) 2.2 ± 1.0	2 (2–3) 2.3 ± 0.9	2 (2–3) 2.2 ± 0.9	0.03

^1^*p* value for difference between patients with and without heart failure.^2^ *p* value for difference between heart failure patients with reduced, mid-range and preserved ejection fraction.^3^ CHA2DS2-VASc score 0 for men and 1 for women.^4^ CHA2DS2-VASc score 1 for men and 2 for women.^5^ CHA2DS2-VASc score ≥ 2 for men and ≥ 3 for women. Abbreviations: See Table 1; OAC, oral anticoagulation.

**Table 3:** (**A**) Proportion of patients receiving oral anticoagulation at hospital admission in relation to heart failure presence and indications to oral anticoagulation [2]. (**B**) Proportion of patients not receiving oral anticoagulants at hospital admission who received oral anticoagulation at discharge in relation to heart failure presence and indications to oral anticoagulation [2].

(A)	No Indications to OAC		Class IIa Indications to OAC		Class I Indications to OAC	
AF/non–HF						
Overall	65 (74%)		135 (78%)		768 (85%)	
-HAS-BLED 0	59 (91%)		3 (2.2%)		1 (0.1%)	
-HAS-BLED 1–2	6 (9.2%)		131 (97%)		593 (77%)	
-HAS-BLED ≥3	0 (0%)		1 (0.7%)		174 (23%)	
AF/HF						
Overall	NA		44 (72%)		2276 (84%)	
-HAS-BLED 0	NA		29 (66%)		15 (0.7%)	
-HAS-BLED 1–2	NA		15 (34%)		1540 (68%)	
-HAS-BLED ≥ 3	NA		0 (0%)		721 (32%)	
**(B)**	**No Indications to OAC**		**Class IIa Indications to OAC**		**Class I Indications to OAC**	
AF/non–HF						
Overall	23 (26%)		39 (22%)		136 (15%)	
-HAS-BLED 0	19 (83%)		3 (7.7%)		1 (0.7%)	
-HAS-BLED 1–2	4 (17%)		35 (90%)		85 (63%)	
-HAS-BLED ≥3	0 (0%)		1 (2.6%)		50 (37%)	
	OAC at discharge	No OAC at discharge	OAC at discharge	No OAC at discharge	OAC at discharge	No OAC at discharge
Overall	10 (43%)	13 (57%)	23 (59%)	16 (41%)	80 (59%)	56 (41%)
-HAS-BLED 0	10 (100%)	9 (69%)	0 (0%)	3 (19%)	0 (0%)	1 (1.8%)
-HAS-BLED 1–2	0 (0%)	4 (31%)	22 (96%)	13 (81%)	57 (71%)	28 (50%)
-HAS-BLED ≥3	0 (0%)	0 (0%)	1 (4.3%)	0 (0%)	23 (29%)	27 (48%)
AF/HF						
Overall	NA		17 (28%)		430 (16%)	
-HAS-BLED 0	NA		13 (76%)		3 (0.7%)	
-HAS-BLED 1–2	NA		3 (15%)		202 (47%)	
-HAS-BLED ≥3	NA		1 (5.9%)		225 (52%)	
	OAC at discharge	No OAC at discharge	OAC at discharge	No OAC at discharge	OAC at discharge	No OAC at discharge
Overall	NA	NA	13 (76%)	4 (24%)	268 (62%)	162 (38%)
-HAS-BLED 0	NA	NA	10 (77%)	3 (75%)	2 (0.7%)	1 (0.6%)
-HAS-BLED 1–2	NA	NA	2 (15%)	1 (25%)	137 (51%)	65 (40%)
-HAS-BLED ≥3	NA	NA	1 (7.7%)	0 (0%)	129 (48%)	96 (59%)

Class IIa indications to OAC: CHA_2_DS_2_-VASc 1 (if male), 2 (if female). Class I indications to OAC: CHA_2_DS_2_-VASc ≥2 (if male), ≥3 (if female). Differences in non-oral anticoagulation prescription at admission between AF/non–HF vs. AF/HF group were not statistically significant for both, class IIa (*p* = 0.39) and class I (*p* = 0.50) indications to oral anticoagulation. Differences in non-oral anticoagulation prescription at discharge between AF/non–HF vs. AF/HF group were not statistically significant for both, class IIa (*p* = 0.54) and class I (*p* = 0.48) indications to oral anticoagulation. Presented data included only patients with information on oral anticoagulation at hospital admission and at hospital discharge (*n* = 3933). Abbreviations: See Table 1.

## Supplementary Materials

The following are available online at https://www.mdpi.com/2077-0383/10/7/1341/s1, Figure S1. Heart rhythm at hospital admission and its changes during hospitalization. Figure S2. Proportion of patients not receiving oral anticoagulation at hospital admission in relation to the presence or absence of heart failure and indications to oral anticoagulation. Figure S3. Prescription rate of heart failure medications in patients with and without heart failure (medication at discharge). Figure S4. Pharmacotherapy of hospitalized atrial fibrillation patients depending on the presence or absence of heart failure and its subtypes. Table S1. Laboratory and echocardiographic parameters of hospitalized atrial fibrillation patients depending on the presence or absence of heart failure and its subtypes. Table S2. Characteristics and treatment of patients receiving vs. not receiving oral anticoagulation at hospital admission. Table S3. Baseline characteristics of atrial fibrillation patients hospitalized in academic and territorial hospitals.
