# Supplementary material for: Characteristics and Treatment of Atrial Fibrillation with Respect to the Presence or Absence of Heart Failure. Insights from the Multicenter Polish Atrial Fibrillation (POL-AF) Registry
Source: J Clin Med. 2021 Mar 24;10(7):1341. doi: 10.3390/jcm10071341 (PMC8036873; doi:10.3390/jcm10071341)
Supplement: Supplementary file 1 [file jcm-10-01341-s001.pdf]

Supplementary Materials

Table S1. Laboratory and echocardiographic parameters of hospitalized atrial fibrillation patients depending on presence or absence of heart failure and its subtypes.

Variable	AF/non–HF (n=1177)	AF/HF (n=2822)	p^1	AF/HF with known EF (n=2726)			p^2
				**HFrEF (n=950)**	**HFmrEF (n=417)**	**HFpEF (n=1359)**	
**Laboratory tests**							
Hemoglobin (g/dl)	13.7 [12.6-14.7] *n=1159*	13.1 [11.8-14.3] *n=2797*	**<0.01**	13.2 [11.8-14.4] *n=944*	13.1 [11.8-14.2] *n=415*	13.1 [11.9-14.2] *n=1342*	0.79
eGFR (ml/min/1.73m^2)	60.0 [53.0-78.5] *n=1121*	59.0 [44.0-75.0] *n=2776*	**<0.01**	56.8 [43.0-69.9] *n=938*	60.0 [45.0-79.9] *n=408*	60.0 [44.9-77.1] *n=1335*	**<0.01**
eGFR<60ml/ min/1.73m^2	701 (38%)	1407 (51%)	**<0.01**	522 (56%)	199 (49%)	639 (48%)	**<0.01**
Glucose (mg/dl)	101.0 [93.0-119.0] *n=915*	104 .0 [93.0-124.0] *n=2064*	0.08	105.0 [92.0-125.0] *n=709*	104.0 [95.0-124.0] *n=293*	103.0 [93.0-122.0] *n=992*	0.63
LDL (mg/dl)	93.0 [63.0-136.0] *n=727*	81.0 [58.0-113] *n=1749*	**<0.01**	81.2 [58.0-117.5] *n=620*	77.0 [56.0-104.0] *n=273*	83.0 [61.0-115.0] *n=814*	0.05
HDL (mg/dl)	48.0 [39.0-58.0] *n=726*	44.0 [36.0-54.0] *n=1754*	**<0.01**	43.7 [35.0-54.5] *n=621*	43.0 [34.4-52.2] *n=274*	45.0 [36.7-54.1] *n=817*	0.10
Triglycerides (mg/dl)	116.0 [62.9-167.0] *n=723*	106.3 [79.7-143.0] *n=1753*	**<0.01**	109.0 [81.0-153.1] *n=622*	107.0 [80.0-140.0] *n=275*	104.3 [76.2-138.0] *n=814*	0.05
**Echocardiography parameters**							
Left ventricular ejection fraction (%)	58.0 [55.0-60.0] *n=760*	50.0 [36.0-58.0] *n=2363*	**<0.01**	30.0 [25.0-37.0] *n=813*	45.0 [40.0-47.0] *n=367*	58.0 [55.0-60.0] *n=1183*	**<0.01**
Left atrial diameter (mm)	43.0 [40.0-47.0] *n=547*	48.0 [43.0-52.0] *n=2006*	**<0.01**	49.0 [45.0-54.0] *n=678*	48.0 [44.0-53.0] *n=299*	46.0 [42.0-51.0] *n=1028*	**<0.01**
Left atrial area (cm^2)	27 [23-32] *n=440*	29 [25-35] *n=1491*	**<0.01**	32 [28-38] *n=792*	29 [25-35] *n=477*	28 [24-33] *n=222*	**<0.01**
Left atrial volume index (ml/m^2)	43 [35-53] *n=210*	57 [44-75] *n=880*	**<0.01**	62 [51-79] *n=543*	51 [47-73] *n=223*	54 [42-73] *n=114*	**<0.01**
LVDD (mm)	49.0 [45.0-53.0] *n=707*	53.0 [48.0-59.0] *n=2250*	**<0.01**	59.0 [53.0-66.0] *n=770*	52.0 [48.0-57.0] *n=348*	50.0 [49.0-55.0] *n=1132*	**<0.01**
IVS (mm)	11.0 [10.0-13.0] *n=705*	11.0 [9.0-11.0] *n=2188*	0.50	11.0 [10.0-12.0] *n=731*	11.0 [10.0-12.0] *n=339*	11.0 [11.0-12.5] *n=1118*	**<0.01**

RVD (mm)	31.0 [28.0-34.0] *n=598*	32.0 [29.0-36.0] *n=1889*	**<0.01**	34.0 [31.0-39.0] *n=599*	32.0 [30.0-37.0] *n=299*	31.0 [28.0-34.0] *n=991*	**<0.01**
Aortic stenosis moderate/severe	34 (3.1%) *n=1098*	205 (7.5%) *n=2736*	**<0.01**	75 (8.1%) *n=921*	29 (7.1%) *n=407*	100 ((7.5%) *n=1332*	0.77
Mitral stenosis moderate/severe	37 (3.4%) *n=1098*	61 (2.2%) *n=2736*	**0.04**	32 (3.5%) *n=921*	9 (2.2%) *n=407*	18 (1.4%) *n=1331*	**<0.01**
Mechanical valve replacement	19 (1.6%) *n=1173*	116 (4.1%) *n=2817*	**<0.01**	35 (3.7%) *n=949*	22 (5.3%)	55 (4.1%)	0.39
- mitral valve replacement	12 (1.0%) *n=1176*	72 (2.6%) *n=2821*	**<0.01**	19 (2.0%)	15 (3.6%)	35 (2.6%)	0.22
- aortic valve replacement	9 (0.8%) *n=1174*	62 (2.2%) *n=2818*	**<0.01**	18 (1.9%) *n=949*	12 (2.9%)	29 (2.1%)	0.51

[1] p value for difference between patients with and without heart failure

[2] p value for difference between heart failure patients with reduced, mid-range and preserved ejection fraction

Abbreviations: AF, atrial fibrillation; eGFR, estimated glomerular filtration rate; HDL, high density lipoprotein; HF, heart failure; HFmEF, heart failure with mild-range ejection fraction; HFpEF, heart failure with preserved ejection fraction; HFrEF, heart failure with reduced ejection fraction; IVS, interventricular septum; LDL, low density lipoprotein; LVDD, left ventricular diastolic dimension; RVD, right ventricular dimension

Table S2. Characteristics and treatment of patients receiving vs not receiving oral anticoagulation at hospital admission.

Variable	AF/non–HF		p^1	AF/HF		p^2
	No OAC (n=200)	OAC (n=971)		No OAC (n=462)	OAC (n=2342)	
**Demographics**						
Age (years)	69 [61-77]	70 [64-79]	**0.02**	75 [68-83]	73 [66-81]	0.11
Females (%)	83 (42%)	453 (47%)	0.18	196 (42%)	962 (41%)	0.61
BMI (kg/m^2)	29 [26-32]	29 [26-31]	0.38	28 [25-32]	29 [26-32]	0.46
**AF type**						
AF paroxysmal	141 (70%)	522 (54%)	**<0.01**	224 (48%)	1029 (44%)	0.07
AF persistent	44 (22%)	291 (30%)	**0.03**	106 (23%)	488 (21%)	0.32
AF permanent	15 (7.5%)	158 (16%)	**<0.01**	132 (29%)	825 (35%)	**<0.01**
**AF history**						
Prior AF history	119 (60%)	919 (95%)	**<0.01**	352 (76%)	2285 (98%)	**<0.01**
Prior DC cardioversion for AF	19 (9.5%)	192 (20%)	**<0.01**	35 (7.6%)	674 (29%)	**<0.01**
Prior ablation for AF	8 (4.0%)	96 (9.9%)	**<0.01**	16 (3.5%)	144 (6.2%)	**0.02**
**Comorbidities**						
Hypertension	144 (72%)	788 (81%)	**<0.01**	374 (81%)	2019 (88%)	**<0.01**
CAD	66 (33%)	300 (31%)	0.56	260 (56%)	1385 (59%)	0.26
PAD	19 (9.5%)	86 (8.9%)	0.79	84 (18%)	385 (16%)	0.38
Valvular disease	78 (39%)	351 (36%)	0.47	282 (61%)	1518 (65%)	0.14
Previous stroke	20 (10%)	97 (10%)	1.00	49 (11%)	329 (14%)	0.052
Thromboembolic events	21 (11%)	127 (13%)	0.35	61 (13%)	444 (20%)	**<0.01**
Hemorrhagic events	21 (11%)	37 (3.8%)	**<0.01**	57 (12%)	136 (5.8%)	**<0.01**
Diabetes mellitus	47 (24%)	269 (28%)	0.26	173 (37%)	870 (37%)	0.92
Chronic kidney disease	21 (11%)	116 (12%)	0.63	153 (33%)	730 (31%)	0.41
Smoking (current/former)	49 (25%)	206 (23%)	0.58	135 (30%)	656 (30%)	0.91
Alcohol overconsumption (≥8 drinks/week)	7 (3.6%)	14 (1.5%)	0.08	29 (6.3%)	99 (4.5%)	0.09
Liver disease	12 (6.0%)	34 (3.5%)	0.11	44 (9.5%)	170 (7.3%)	0.10
Thyroid disease	27 (14%)	177 (18%)	0.12	73 (16%)	443 (19%)	0.13
COPD/asthma	11 (5.5%)	56 (5.8%)	1.00	59 (13%)	322 (14%)	0.60
**Laboratory tests**						
Hemoglobin (g/dl)	13.7 [12.4-15.0]	13.7 [12.6-14.6]	0.45	12.8 [11.3-14.1]	13.2 [12.0-14.3]	**<0.01**
eGFR (ml/min)	67 [56-90]	60 [52-76]	**<0.01**	57 [40-78]	60 [45-75]	**0.17**
**Antiplatelet therapy at hospital admission**						
Any antiplatelet therapy	45 (23%)	67 (6.9%)	**<0.01**	157 (34%)	322 (14%)	**<0.01**
Single antiplatelet therapy	34 (17%)	36 (3.7%)	**<0.01**	100 (22%)	206 (8.8%)	**<0.01**

Dual antiplatelet therapy	11 (5.5%)	31 (3.2%)	0.14	57 (12%)	116 (5.0%)	**<0.01**
**Thromboembolic and bleeding risk**						
CHA2DS2-VASc score	3 [2-4]	3 [2-4]	**<0.01**	5 [4-6]	5 [4-6]	**<0.01**
HAS-BLED score	2 [1-3]	2 [1-3]	0.73	3 [2-3]	2 [2-3]	**<0.01**
**Oral anticoagulation at hospital discharge**						
None	83 (42%) *n=198*	17 (1.8%) *n=968*	**<0.01**	166 (37%) *n=447*	71 (3.1%) *n=2320*	**<0.01**
VKA	7 (3.5%) *n=198*	144 (15%) *n=968*	**<0.01**	24 (5.2%)	464 (20%)	**<0.01**
rivaroxaban	41 (21%) *n=198*	356 (37%) *n=968*	**<0.01**	72 (16%)	726 (31%)	**<0.01**
dabigatran	16 (8.1%) *n=198*	250 (26%) *n=968*	**<0.01**	51 (11%)	501 (22%)	**<0.01**
apixaban	51 (26%) *n=198*	201 (21%) *n=968*	0.13	134 (30%)	558 (24%)	**0.01**
**OAC transition**						
VKA to NOAC	NA	15 (1.5%)	NA	NA	66 (2.8%)	NA
VKA to rivaroxaban	NA	1 (0.1%)	NA	NA	18 (0.8%)	NA
VKA to dabigatran	NA	3 (0.3%)	NA	NA	11 (0.5%)	NA
VKA to apixaban	NA	11 (1.1%)	NA	NA	37 (1.6%)	NA
NOAC* to VKA	NA	2 (0.2%)*	NA	NA	12 (0.5%)**	NA
Rivaroxaban to dabigatran	NA	2 (0.2%)	NA	NA	6 (0.3%)	NA
Rivaroxaban to apixaban	NA	4 (0.4%)	NA	NA	41 (1.8%)	NA
Dabigatran to rivaroxaban	NA	2 (0.2%)	NA	NA	3 (0.1%)	NA
Dabigatran to apixaban	NA	1 (0.1%)	NA	NA	16 (0.7%)	NA

[1] p value for difference between patients with and without heart failure

[2] p value for difference between heart failure patients with reduced, mid-range and preserved ejection fraction

*dabigatran

**dabigatran (0.1%), rivaroxaban (0.3%), apixaban (0.2%)

Abbreviations: AF, atrial fibrillation; BMI, body mass index; CAD, coronary artery disease; COPD, chronic obstructive pulmonary disease; DC, direct current; eGFR, estimated glomerular filtration rate; EHRA, European Heart Rhythm Association; HF, heart failure; HFmEF, heart failure with mild-range ejection fraction; HFpEF, heart failure with preserved ejection fraction; HFrEF, heart failure with reduced ejection fraction; NA, non-applicable; NYHA, New York Heart Association; PAD, peripheral artery disease

Table S3. Baseline characteristics of atrial fibrillation patients hospitalized in academic and territorial hospital.

Variable	Patients from academic hospital (n=3396)	Patients from tertiary hospital (n=603)	p[1]
**Demographics**			
Age (years)	72 [65-81]	73 [67-82]	**0.01**
Females (%)	1427 (42%)	277 (46%)	0.07
BMI (kg/m^2)	28 [26-32]<br>*n=2572*	29 [27-34]<br>*n=170*	**<0.01**
**Primary reason of index hospital admission**			
AF without any procedures	190 (5.6%)	62 (10%)	**<0.01**
DC cardioversion for AF	793 (23%)	100 (17%)	**<0.01**
HF decompensation	581 (17%)	225 (37%)	**<0.01**
Elective CIED implantation/ replacement	298 (8.8%)	62 (10%)	0.25
ACS	236 (7.0%)	11 (1.8%)	**<0.01**
Elective PCI	374 (11%)	9 (1.5%)	**<0.01**
Non-AF-ablation	200 (5.9%)	10 (1.7%)	**<0.01**
Other	717 (21%)	112 (19%)	**<0.01**
**AF type**			
AF paroxysmal	1660 (49%)	263 (44%)	**0.02**
AF persistent	765 (23%)	168 (28%)	**<0.01**
AF permanent	971 (29%)	172 (29%)	1.00
**AF history**			
Prior AF history	3138 (92%)	559 (93%)	**0.87**
Prior DC cardioversion for AF	821 (24%)	99 (16%)	**<0.01**
Prior AF-ablation	226 (6.7%)	38 (6.3%)	0.79
EHRA I	1306 (54%)<br>*n=2411*	49 (13%)<br>*n=369*	**<0.01**
EHRA II	720 (30%)<br>*n=2411*	247 (67%)<br>*n=369*	**<0.01**
- EHRA IIa	348 (14%)<br>*n=2409*	46 (12%)<br>*n=369*	0.34
- EHRA IIb	289 (13%)<br>*n=2409*	47 (13%)<br>*n=369*	0.67
EHRA III	313 (14%)<br>*n=2411*	64 (17%)<br>*n=369*	**0.03**
EHRA IV	72 (3.0%)<br>*n=2411*	9 (2.4%)<br>*n=369*	0.74
**HF**			
HF	2480 (73%)	342 (57%)	**<0.01**
Previous HF diagnosis	2291 (67%)	330 (55%)	**<0.01**
HF de novo	189 (5.6%)	12 (2.0%)	**<0.01**
**Comorbidities**			
Hypertension	2853 (84%)	491 (81%)	0.12
Vascular disease	1996 (59%)	249 (41%)	**<0.01**
Previous stroke	424 (12%)	76 (13%)	0.95
Thromboembolic events	548 (16%)	111 (18%)	0.17
Hemorrhagic events	222 (6.5%)	29 (4.8%)	0.12
Diabetes mellitus	1153 (34%)	213 (35%)	0.51
Chronic kidney disease	866 (26%)	163 (27%)	0.45
Smoking (current/former)	855 (27%)<br>*n=3218*	196 (35%)<br>*n=557*	**<0.01**
Alcohol overconsumption ($\geq$8 drinks/week)	128 (4.0%)<br>*n=3206*	22 (3.7%)<br>*n=602*	0.82
Liver disease	241 (7.1%)	20 (3.3%)	**<0.01**
Thyroid disease	608 (18%)	119 (20%)	0.28
COPD/asthma	372 (11%)	76 (13%)	0.23
Device therapy*	794 (23%)	85 (14%)	**<0.01**
CHA_2DS_2-VASc score	5 [3-6]	4 [3-5]	**<0.01**

	4.4+1.8	4.3±1.8	
HAS-BLED score	2 [2-3]<br>2.1±1.0	2 [1-2]<br>2.1±1.0	**<0.01**
**Medications at hospital admission**			
No OAC	573 (17%)<br>*n=3374*	89 (15%)<br>*n=601*	0.21
No OAC despite class I recommendations	509 (16%)<br>*n=3098*	74 (13%)<br>*n=553*	0.08
VKA	667 (20%)<br>*n=3374*	48 (8.0%)<br>*n=601*	**<0.01**
Dabigatran	623 (18%)<br>*n=3374*	142 (24%)<br>*n=601*	**<0.01**
Rivaroxaban	990 (29%)<br>*n=3374*	165 (27%)<br>*n=601*	0.35
Apixaban	521 (15%)<br>*n=3374*	157 (26%)<br>*n=601*	**<0.01**
Antiplatelet drugs	565 (17%)<br>*n=3371*	27 (4.5%)<br>*n=601*	**<0.01**
**Medications at hospital discharge**			
No OAC	300 (8.9%)<br>*n=3354*	45 (7.5%)<br>*n=602*	0.27
No OAC despite class I recommendations	269 (8.7%)<br>*n=3079*	41 (7.4%)<br>*n=554*	0.32
VKA	603 (18%)<br>*n=3354*	37 (6.1%)<br>*n=602*	**<0.01**
Dabigatran	661 (20%)<br>*n=3354*	158 (26%)<br>*n=602*	**<0.01**
Rivaroxaban	1028 (31%)<br>*n=3354*	171 (28%)<br>*n=602*	0.29
Apixaban	762 (23%)<br>*n=3354*	191 (32%)<br>*n=602*	**<0.01**
Antiplatelet drugs	511 (15%)<br>*n=3370*	19 (3.2%)<br>*n=601*	**<0.01**
Beta-blockers	2906 (87%)	492 (82%)	**<0.01**
Digoxin	261 (7.8%)<br>*n=3351*	56 (9.3%)<br>*n=602*	0.22
NdhpCCB	24 (0.7%)<br>*n=3351*	3 (0.5%)<br>*n=602*	1.00
Amidarone	554 (17%)<br>*n=3351*	197 (33%)<br>*n=602*	**<0.01**
AAD I class	359 (11%)<br>*n=3350*	24 (4.0%)<br>*n=602*	**<0.01**
AAD I class in patients with HFpEF	195 (16%)<br>*n=1219*	7 (5.3%)<br>*n=131*	**<0.01**
RAS inhibitors	2672 (79%)	430 (71%)	**<0.01**
DhpCCB	1044 (31%)<br>*n=3351*	220 (37%)<br>*n=602*	**0.01**
MRA	1338 (40%)<br>*n=3351*	262 (44%)<br>*n=602*	0.10
Diuretics	2125 (63%)<br>*n=3351*	441 (73%)<br>*n=602*	**<0.01**

Abbreviations: AAD, antiarrhythmic drug; AF, atrial fibrillation; BMI, body mass index; CAD, coronary artery disease; COPD, chronic obstructive pulmonary disease; DC, direct current; dhpCCB, dihydropyridine calcium channel blockers; EHRA, European Heart Rhythm Association; HF, heart failure; HFmEF, heart failure with mild-range ejection fraction; HFpEF, heart failure with preserved ejection fraction; HFrEF, heart failure with reduced ejection fraction; MRA, mineralocorticoid receptor antagonist; ndhpCCB, non-dihydropyridine calcium channel blockers; NYHA, New York Heart Association; OAC, oral anticoagulation; PAD, peripheral artery disease; RAS, renin-angiotensin system; VKA, vitamin K antagonist

Figure S1. Heart rhythm at hospital admission and its changes during hospitalization.

a) Proportion of patients in atrial fibrillation and those in sinus rhythm at hospital admission with regard to presence or absence of heart failure

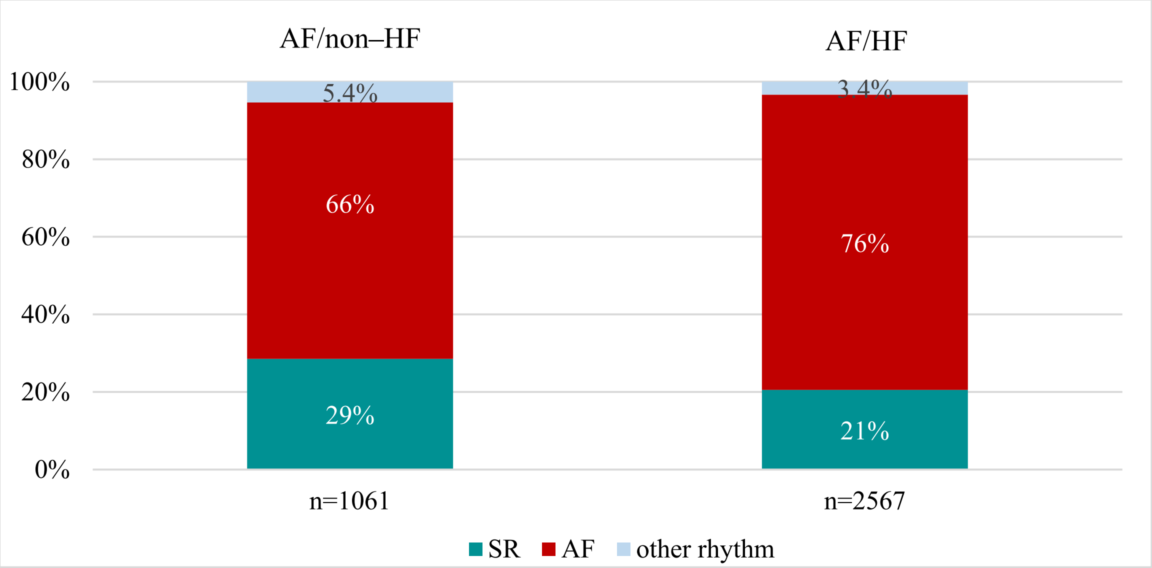

Differences between AF/non–HF vs AF/HF group were statistically significant.

Presented data included only patients with information on heart rhythm on hospital admission.

b) Proportion of patients who converted from atrial fibrillation to sinus rhythm during hospitalization and those who remained in sinus rhythm throughout hospitalization with regard to presence or absence of heart failure

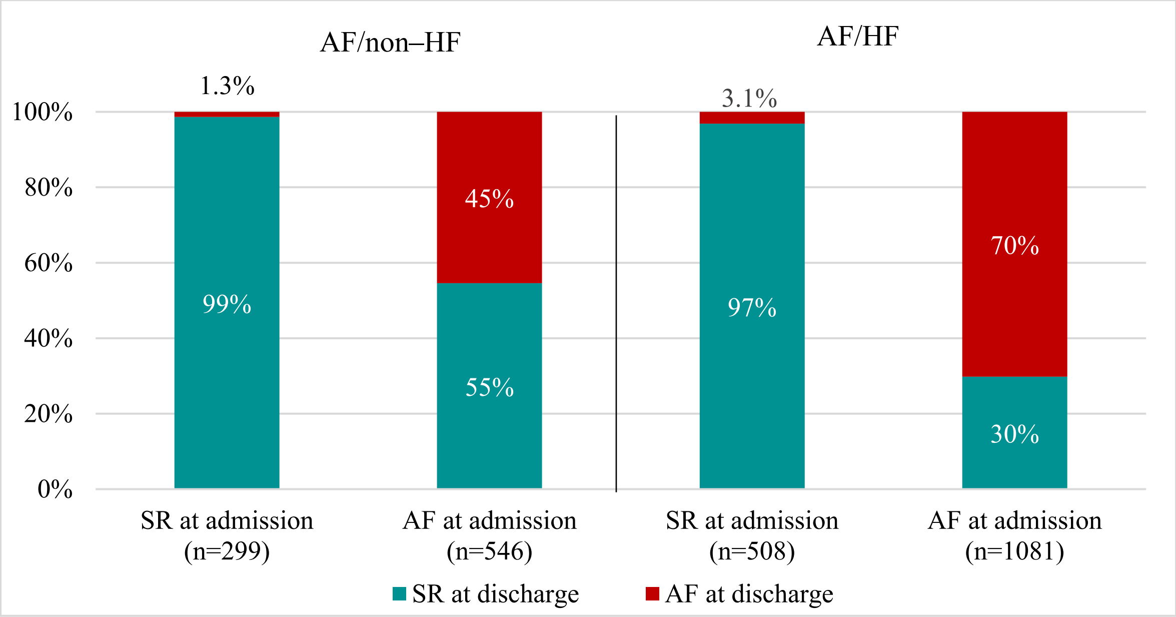

Differences between AF/non–HF vs AF/HF group were statistically significant.

Presented data included only patients with simultaneous information on heart rhythm both on admission and at discharge.

Abbreviations: See **Table 1**; SR, sinus rhythm

Figure S2. Proportion of patients not receiving oral anticoagulation at hospital admission in relation to presence or absence of heart failure and indications to oral anticoagulation. (2)

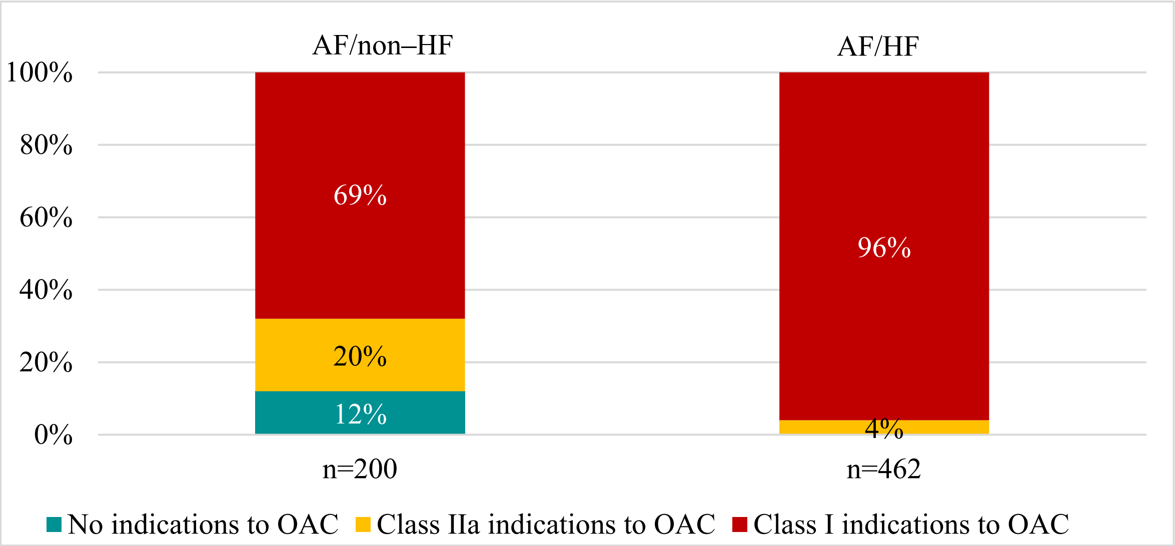

Abbreviations: See **Figure 4**

Figure S3. Prescription rate of heart failure medications in patients with and without heart failure (medication at discharge)**.**

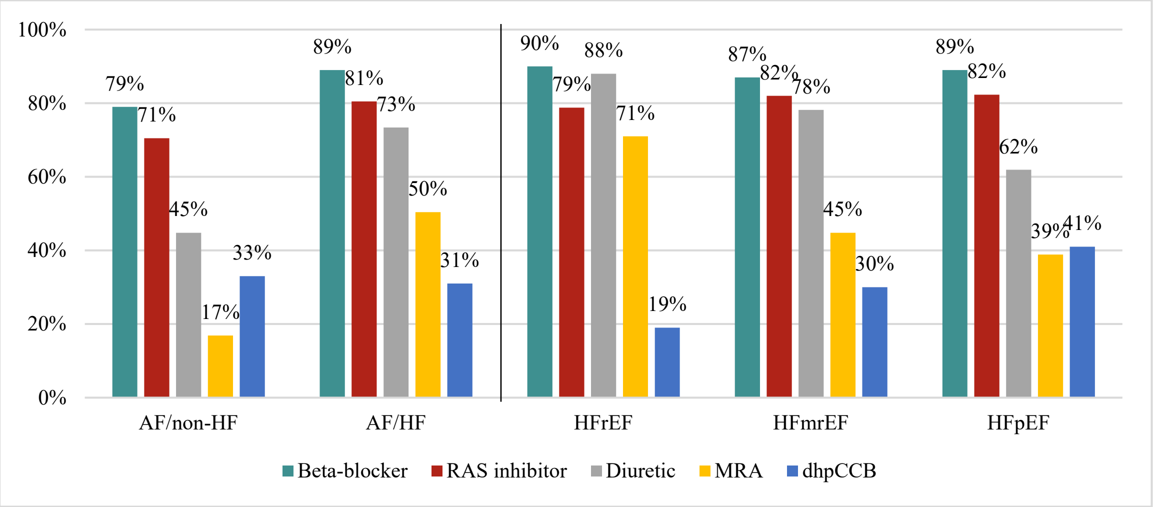

Differences in pharmacotherapy between AF/nonHF vs AF/HF group were statistically significant ($p<0.05$), expect dihydropyridine calcium channel blocker treatment ($p=0.27$).

Differences in pharmacotherapy between HFrEF, HFmrEF and HFrEF groups were statistically significant ($p<0.05$), except expect RAS inhibitor ($p=0.11$) and beta-blocker ($p=0.26$) treatment.

Abbreviations: See **Table 1**; nhpCCB, dihydropyridine calcium channel blocker; MRA, mineralocorticoid receptor antagonist; RAS, renin-angiotensin system

Figure S4. Pharmacotherapy of hospitalized atrial fibrillation patients depending on presence or absence of heart failure and its subtypes.

a) Anticoagulation

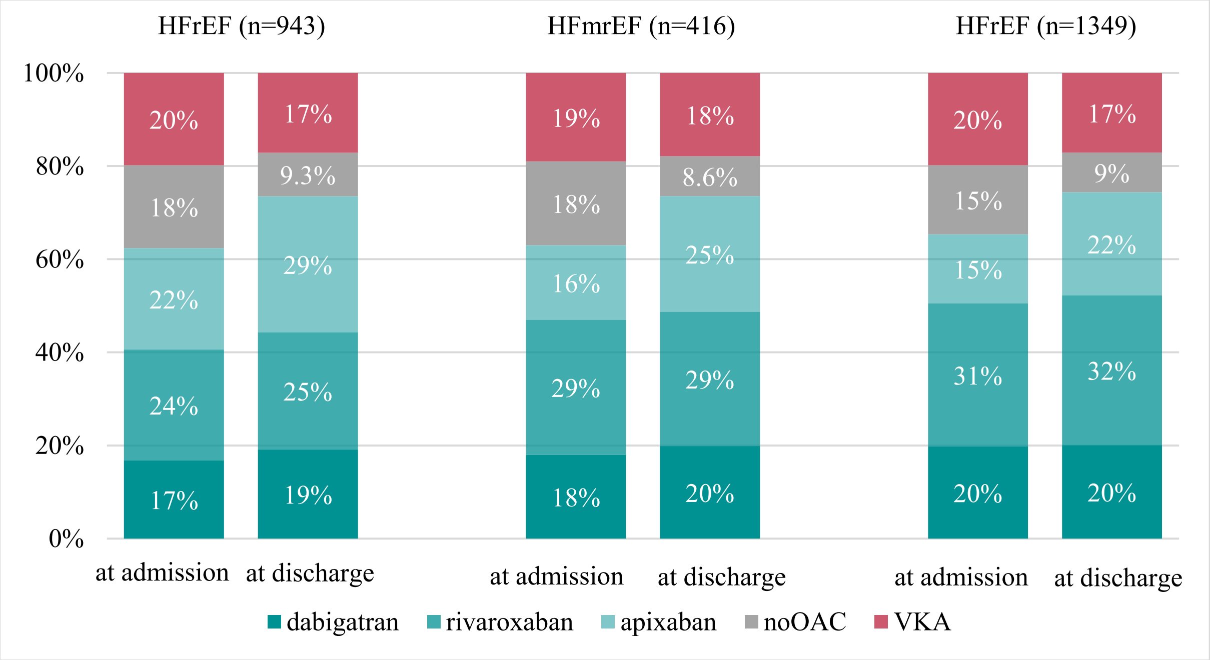

There were statistically significant differences ($p<0.05$) between HFrEF, HFmrEF and HFpEF groups with regard to treatment with rivaroxaban and apixaban (both at baseline and at discharge).

b) NOAC standard and reduced doses

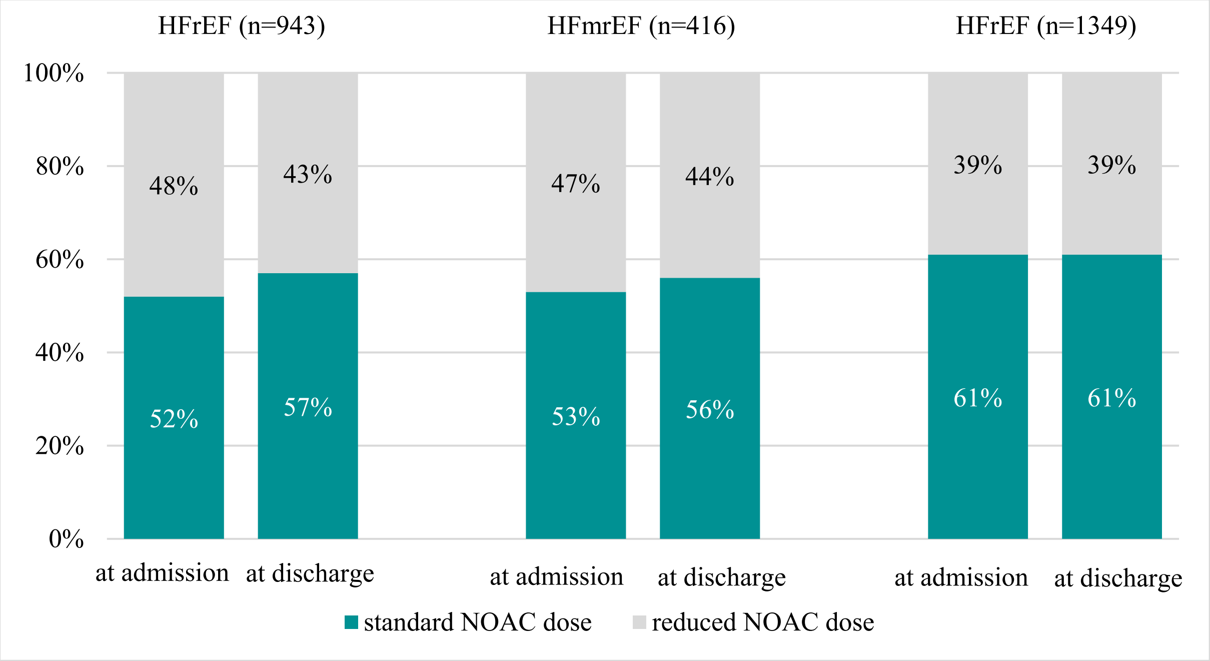

Differences between HFrEF, HFmrEF and HFpEF groups with regard to reduction in NOAC doses at baseline were statistically significant ($p<0.05$).

Abbreviations: See **Table 1**; NOAC, non-vitamin K antagonist oral anticoagulant

c) rate and rhythm controlling drugs at discharge

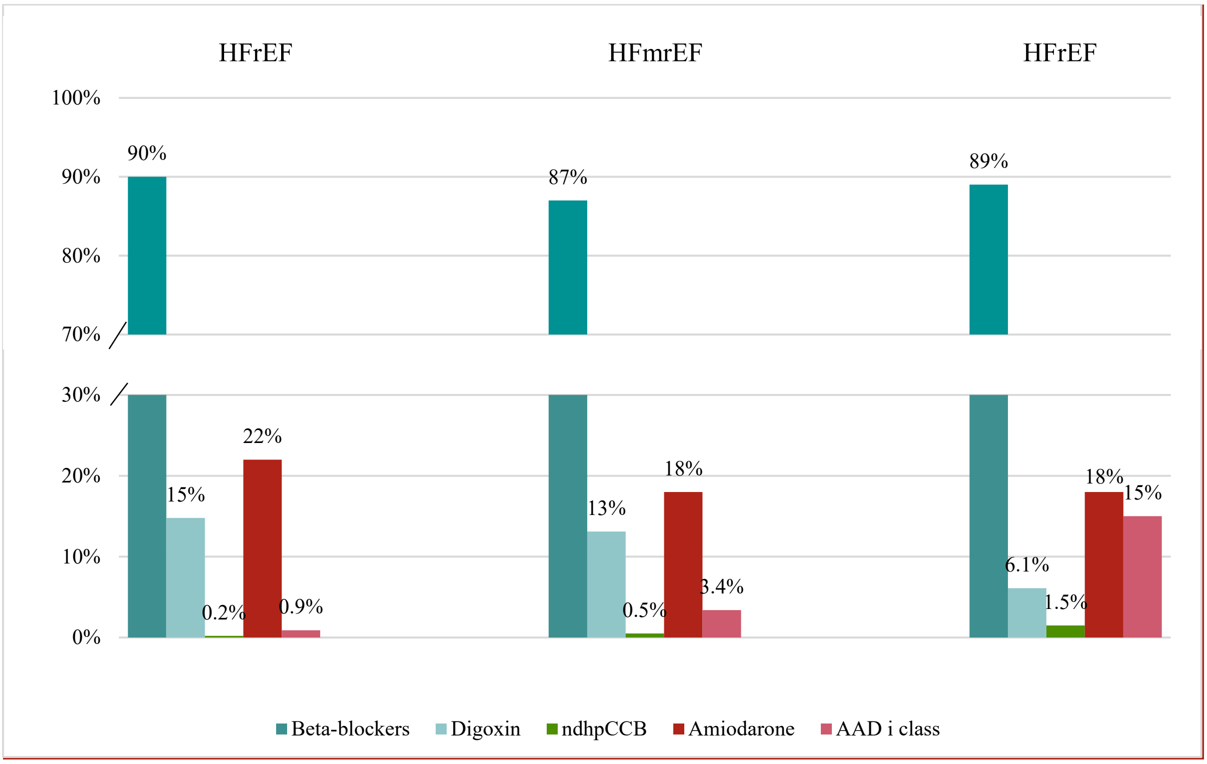

Dronedarone was not prescribed in any of the groups.

Differences in pharmacotherapy between HFrEF, HFmrEF and HFrEF groups were statistically significant ($p<0.05$), except beta-blockers treatment ($p=0.26$). **Abbreviations:** See **Table 1**; AAD, antiarrhythmic drug; ndhpCCB, non-dihydropyridine calcium channel blockers
